# The Deleterious Effects of Impaired Fibrinolysis on Skeletal Development Are Dependent on Fibrin(ogen), but Independent of Interlukin-6

**DOI:** 10.3389/fcvm.2021.768338

**Published:** 2021-12-06

**Authors:** Heather A. Cole, Stephanie N. Moore-Lotridge, Gregory D. Hawley, Richard Jacobson, Masato Yuasa, Leslie Gewin, Jeffry S. Nyman, Matthew J. Flick, Jonathan G. Schoenecker

**Affiliations:** ^1^Departments of Nuclear Medicine, Vanderbilt University Medical Center, Nashville, TN, United States; ^2^Departments of Orthopaedics, Vanderbilt University Medical Center, Nashville, TN, United States; ^3^Center of Bone Biology, Vanderbilt University Medical Center, Nashville, TN, United States; ^4^Department of Pharmacology, Vanderbilt University, Nashville, TN, United States; ^5^Department of Orthopaedic and Spinal Surgery, Tokyo Medical and Dental University, Tokyo, Japan; ^6^Departments of Medicine, Vanderbilt University Medical Center, Nashville, TN, United States; ^7^Department of Research, Veterans Affairs, Tennessee Valley Healthcare System, Nashville, TN, United States; ^8^Department of Biomedical Engineering, Vanderbilt University, Nashville, TN, United States; ^9^Department of Pathology and Laboratory Medicine, University of North Carolina, Chapel Hill, NC, United States; ^10^University of North Carolina Blood Research Center, University of North Carolina, Chapel Hill, NC, United States; ^11^Lineberger Comprehensive Cancer Center, University of North Carolina, Chapel Hill, NC, United States; ^12^Departments of Pathology, Microbiology and Immunology, Vanderbilt University Medical Center, Nashville, TN, United States; ^13^Departments of Pediatrics, Vanderbilt University Medical Center, Nashville, TN, United States

**Keywords:** fibrinolysis, plasminogen, bone development, skeletal development, interlukin-6, fibrin, fibrinogen

## Abstract

Chronic diseases in growing children, such as autoimmune disorders, obesity, and cancer, are hallmarked by musculoskeletal growth disturbances and osteoporosis. Many of the skeletal changes in these children are thought to be secondary to chronic inflammation. Recent studies have likewise suggested that changes in coagulation and fibrinolysis may contribute to musculoskeletal growth disturbances. In prior work, we demonstrated that mice deficient in plasminogen, the principal protease of degrading and clearing fibrin matrices, suffer from inflammation-driven systemic osteoporosis and that elimination of fibrinogen resulted in normalization of IL-6 levels and complete rescue of the skeletal phenotype. Given the intimate link between coagulation, fibrinolysis, and inflammation, here we determined if persistent fibrin deposition, elevated IL-6, or both contribute to early skeletal aging and physeal disruption in chronic inflammatory conditions. Skeletal growth as well as bone quality, physeal development, and vascularity were analyzed in C57BL6/J mice with plasminogen deficiency with and without deficiencies of either fibrinogen or IL-6. Elimination of fibrinogen, but not IL-6, rescued the skeletal phenotype and growth disturbances in this model of chronic disease. Furthermore, the skeletal phenotypes directly correlated with both systemic and local vascular changes in the skeletal environment. In conclusion, these results suggest that fibrinolysis through plasmin is essential for skeletal growth and maintenance, and is multifactorial by limiting inflammation and preserving vasculature.

## Summary

Elimination of plasmin-mediated fibrin clearance impairs skeletal development and systemic vascularity, independent of IL-6. Fibrinogen-deficiency rescued skeletal development and systemic vascularity in plasminogen-deficient mice.

## Introduction

Children with chronic disease such as malnutrition, obesity, infection, cancer, cytotoxic chemotherapy, or autoimmune disease have all been shown to experience growth disturbances and osteoporosis (1–7). Recent studies have suggested that hallmarks of chronic disease, such as systemic inflammation, aberrations in coagulation, and vascular dysfunction contribute to abnormal skeletal development and growth disturbances. Later in life, perturbed skeletal development can have devastating consequences for both appendicular and axial length, as well as angular deformities which together can significantly alter a child's mobility and quality of life.

Chronic inflammation is considered by most to be a primary contributor to the skeletal disturbances observed during development. For example, mice that overexpress IL-6, the central pro-inflammatory cytokine, develop a skeletal phenotype that closely resembles the growth and skeletal abnormalities observed in children with chronic inflammatory diseases (2, 8). These studies highlight that chronic elevation of IL-6 results in uncoupling of osteoblast/osteoclast function, delayed ossification, and stunted growth. Beyond measures of inflammation, other studies have suggested that altered coagulation and fibrinolysis may contribute to growth disturbances and early aging, given the observation that there is a considerable imbalance of fibrinogen production and fibrin deposition in chronic diseases (9). Corroborating these findings, prior work from our group demonstrated that mice deficient in plasminogen, the principal protease that removes fibrin, suffer from persistent fibrin deposition within the skeleton and inflammation-driven systemic osteoporosis (10). In these studies, elimination of fibrinogen resulted in normalization of IL-6 levels and complete rescue of the skeletal phenotype. Together, these studies demonstrate an intimate link between coagulation, fibrinolysis, and inflammation.

The overarching goal of this present study was to examine the specific molecular and cellular mechanisms for skeletal disturbances in chronic inflammatory disease. Specifically, we set out to determine if persistent fibrin deposition, elevated IL6, or both were mediators of early skeletal aging and physeal disruption in a chronic inflammatory model. To examine this, we measured skeletal growth and analyzed bone quality and physeal development in mice with impaired fibrinolysis (plasminogen deficiency) superimposed with deficiencies of either fibrinogen or IL-6.

## Materials and Methods

### Animals

Male mice were housed at 22–24°C with a 12-h light/dark cycle and given standard mouse chow (5LOD) and water *ad libitum*. Animals were monitored by the Division of Animal Care (DAC) and all interventions were conducted according to DAC recommendations. Wild type (WT), plasminogen-deficient (*Plg*^−/−^), and plasminogen-deficient/fibrinogen-deficient (*Plg*^−/−^*Fbg*^−/−^) mice on C57BL/6J background were sacrificed at 5, 10, 13, 15 or 20 weeks of age by CO_2_ inhalation. The left femur and spine were placed in 10% formalin for 24 h, examined by micro-computed tomography (μCT), demineralized in 10% EDTA for 7 days and then processed for histology. Kidneys were collected in 10% formalin for 24 h and processed for histology. Interleukin-6-deficient (*IL-6*^−/−^) and plasminogen-deficient/interleukin-6-deficient (*Plg*^−/−^*IL-6*^−/−^) mice on C57BL/6J background were measured longitudinally for weight from 3 to 13 weeks and by radiographs at 8, 10, and 12 weeks of age (Supplementary Table 1). Animal care protocols were approved by the Vanderbilt University Medical Center Institutional Animal Care and Use Committee.

### Embryology

Standard skeletal preparation with alcian blue and alizarin red staining were performed on post-natal day 1 (P1). Skin and viscera were removed and skeletons were fixed in 95% EtOH for 24 h. Following fixation, skeletons were stained in alcian blue for 24 h, placed in 95% EtOH for 3 h, 2% KOH for 24 h, and stained in alizarin red for 24 h. Skeletons were cleared in 1% KOH, placed in 20% glycerol for 48 h, and stored in a 1:1 mixture of glycerol and 95% EtOH.

### Radiographic Skeletal Measurement

To allow for longitudinal measurement of appendicular skeletal growth radiographic analysis was used in place of manual endpoint analysis. WT, *Plg*^−/−^, and *Plg*^−/−^ *Fbg*^−/−^ mice were radiographed weekly from 3 to 20 weeks of age. *IL6*^−/−^ and *Plg*^−/−^ *IL6*^−/−^ were radiographed at 8, 10, and 12 weeks of age. Images were taken for 4 s at 35 kV with a Faxitron LX-60 X-Ray (Lincolnshire, IL). To facilitate congruency between posterioranterior (PA) radiographs, films were taken with mice in the prone position with hips in abduction, causing external rotation of the tibia, and in the lateral position natural to the mouse (Supplementary Figure 1A). Individual measurements of appendicular (tibia) and axial (spine) length were determined using Image-J (NIH). For longitudinal growth, we first confirmed the absence of curvature in the coronal plane using PA films of prone mice. Spines were then measured in the lateral position from the axis vertebrae (C1) to the superior aspect of the sacrum using the segmented line tool. Tibias were measured from the proximal plateau to the distal plafond using the straight-line tool (Supplementary Figure 1B).

### Manual Skeletal Measurement

Manual measurements were performed to validate our radiographic skeletal measurement technique. Axial skeletal length was measured using calipers to determine nose-to-anus length as previously described (11). Tibial length was measured *ex vivo* following sacrifice. For manual axial measurements, mice were anesthetized (3% isoflurane inhalation for 5 min with constant flow O_2_ at 3 L/min), placed in the prone position, and measured in a straight line from the tip of the nose to the anus using calipers placed parallel and posterior to the spinal column (Supplementary Figure 1C). Caliper measurements of the tibia were made from the distal and proximal articular surfaces (Supplementary Figure 1C). To validate our method of radiographic skeletal measurement, caliper measurements of the axial and appendicular skeleton were then plotted against radiographic measurements (Supplementary Figures 1D,G). The slope of linear regression for tibial length (radiograph vs. caliper) was not significantly different than 1 (1.02 ± 0.06; *p* = 0.80; *R*^2^ = 0.91), indicating that manual measurements of tibial length were strongly correlated radiographically. The slope of the linear regression for axial length (radiograph vs. caliper) was, however, significantly < 1 (0.53 ± 0.04; *p* < 0.0001). Lateral radiographic measurements of the axial skeleton were markedly shorter than caliper measurements because of extraspinal lengths (skull and sacral lengths) included in caliper measurements. Although lateral radiographic measurements could account for kyphosis and lordosis (indicators of changes in spinal length due to degeneration) that were absent in caliper measurements, the variation in extraspinal lengths due to caliper measurements resulted in a slope of the linear regression significantly different that 1. However, because lateral radiographs and manual caliper measurements were strongly correlated (*R*^2^ = 0.92), we conclude that radiographic measurements are a reliable indicator of differences in axial skeletal length.

### Growth Rates

Growth rates were generated following skeletal and weight measurements and designated by three distinct periods of growth based on landmarks in sexual and skeletal development. We have previously demonstrated that the physis of the proximal femur becomes disorganized at 12 weeks of age, indicative of physeal senescence, which is followed by rapid and consistent physeal closure at 13 weeks of age (12). Therefore, these data delineate three separate growth phases: (1) the period of rapid growth preceding sexual maturity (immature growth phase; 3–6 weeks of age), (2) continuous slow growth commencing at puberty and preceding physeal closure (sexually mature growth phase; 7–12 weeks of age), and (3) the period commencing with physeal closure (skeletally mature growth phase; 13–20 weeks of age) (Supplementary Table 2).

### *In vivo* Calcein Labeling and Calculation of Bone Formation Rate

WT and *Plg*^−/−^ mice were injected IP with 200 uL of 0.5% solution of calcein in a 2% sodium bicarbonate solution at 10 and 3 days prior to sacrifice at 10 weeks of age. Mice were sacrificed by CO_2_ inhalation and femurs were collected, fixed, and embedded in methyl methacrylate prior to sectioning at 4 microns. Analysis was performed in a blinded manner using a fluorescent microscope (Olympus BX41, Olympus America, Center Valley, PA) and Osteomeasure software (OsteoMetrics, Decatur, GA). Mineralizing surface per bone surface (MS/BS) represents the percentage of bone surface undergoing active mineralization as determined by the double label surface plus half of the single label surface [MS = (dLS + sLS/2)/BS]. The bone formation rate per bone surface (BFR/BS) was calculated as the product of mineral apposition rate (MAR) and MS/BS as described above. In addition to the aforementioned parameters, bone volume (BV), tissue volume (TV), trabecular thickness (Tb.Th), trabecular number (Tb.N), and trabecular space (Tb.Sp) were measured directly utilizing the Osteomeasure Software.

### Histologic Analysis- Paraffin Embedded Samples

Samples were fixed in 10% formalin and processed to paraffin. Six μm sections were then prepared for staining. Routine hematoxylin and eosin (H&E), martius scarlet blue, Alcian blue, Masson's trichrome, Toluidine blue, and tartrate resistant acid phosphatase stains (TRAP) (Sigma Aldrich, 387A, St. Louis, MO) were performed. The number of osteoblasts (Toluidine blue stain) and osteoclasts (TRAP stain) were quantified in a blinded manner with the Osteomeasure Software. Briefly, osteoblast number (N.Ob), the osteoblast surface per bone surface (Ob.S/BS), and the osteoblast number per bone perimeter (N.Ob/B.Pm) were quantified in 10-week-old WT and *Plg*^−/−^ male following Toludine blue staining. Osteoclast number (N.Oc), the osteoclast surface per bone surface (Oc.S/BS), and the osteoclast number per bone perimeter (N.Oc/B.Pm) were quantified in 10 week old WT and *Plg*^−/−^ male mice following TRAP staining. Immunohistochemistry for VEGF-A (1:100; Abcam, Cambridge, MA) was performed using a standard avidin-biotin-peroxidase-based protocol (Envision+ HRP/DAB System, Dako, North America, Inc., Carpinteria, CA) per manufacturer's instructions. Immunofluorescence for fibrin was performed using a 1:1,000 dilution of rabbit anti-mouse fibrin(ogen) antibody. Slides were then washed with TBS, incubated with 10 μg/mL of Alexa Fluor 647-labeled anti-rabbit antibody (Life Technologies 792514, Grand Island, NY), and counterstained with DAPI. Slides were cover slipped using an aqueous mount (PolySciences, Warrington, PA). Histological sections of the physes were collected from five mice per genotype at each time point (5, 10, and 15 weeks of age), with each section analyzed in three distinct locations (15 images per genotype per time point).

### μCT

Femurs were placed in a standard tube (diameter 12.3 mm) and aligned with the scanning axis of a μCT (μCT40; Scanco Medical; Switzerland). Prior to image acquisition, a region of interest was defined as the area containing the metaphysis, physis, and epiphysis of the distal and proximal femurs. Images were acquired at 55 kVp and 145 μA with a 12 μm isotropic voxel size. After reconstruction, the mean bone volume density of the ossification center in the proximal femoral epiphysis was calculated using a threshold value of 438.7 mgHA/cm^3^ with Scanco Medical evaluation software. The manufacturer's beam hardening correction and calibration phantom of hydroxyapatite (HA) were used to facilitate reconstructions.

### Vascular Casts

Microfil, a radio-opaque silicone rubber compound containing lead chromate (MV-122; Flow Tech Inc., Boulder, CO), was used as previously described (13–15). Femurs and kidneys dissected from mice sacrificed at 5, 10, and 20 weeks of age (*N* = 5 for each age group) were fixed for 48 h in 10% buffered formalin to ensure complete tissue fixation. Angiographic images were obtained using plain radiography and μCT (12-μM isotropic voxel size) as previously described (12).

### Statistical Analysis

Growth velocities from 3–6, 7–12, and 13–20 weeks of age were generated using segmental linear regression. Statistical differences in the growth velocities between groups were compared using analysis of covariance (ANCOVA). If the difference between slopes was found to be statistically non-significant, further analysis comparing the intercepts of the growth velocity curve was provided to fully characterize the differences in the lines. A two-way ANOVA with a Sidak correction was used to determine whether significant differences in the width of physeal zones existed between WT and *Plg*^−/−^ mice across several age groups. Quantification of the ossification of the proximal femoral epiphysis was conducted using Fisher's exact test. Quantification of the bone formation analysis, osteoblast, and osteoclast parameters were conducted using unpaired *t*-tests with multiple comparisons and a false discovery rate of 5%. Corrected *P*-values are reported (^*^p < 0.05). Statistical analyses were performed using GraphPad Prism v6 (GraphPad Software, La Jolla, CA) at α < 0.05.

## Results

### Reduced Fibrinolysis Results in Early Postnatal Cessation of Skeletal Growth

**Figure 1:**
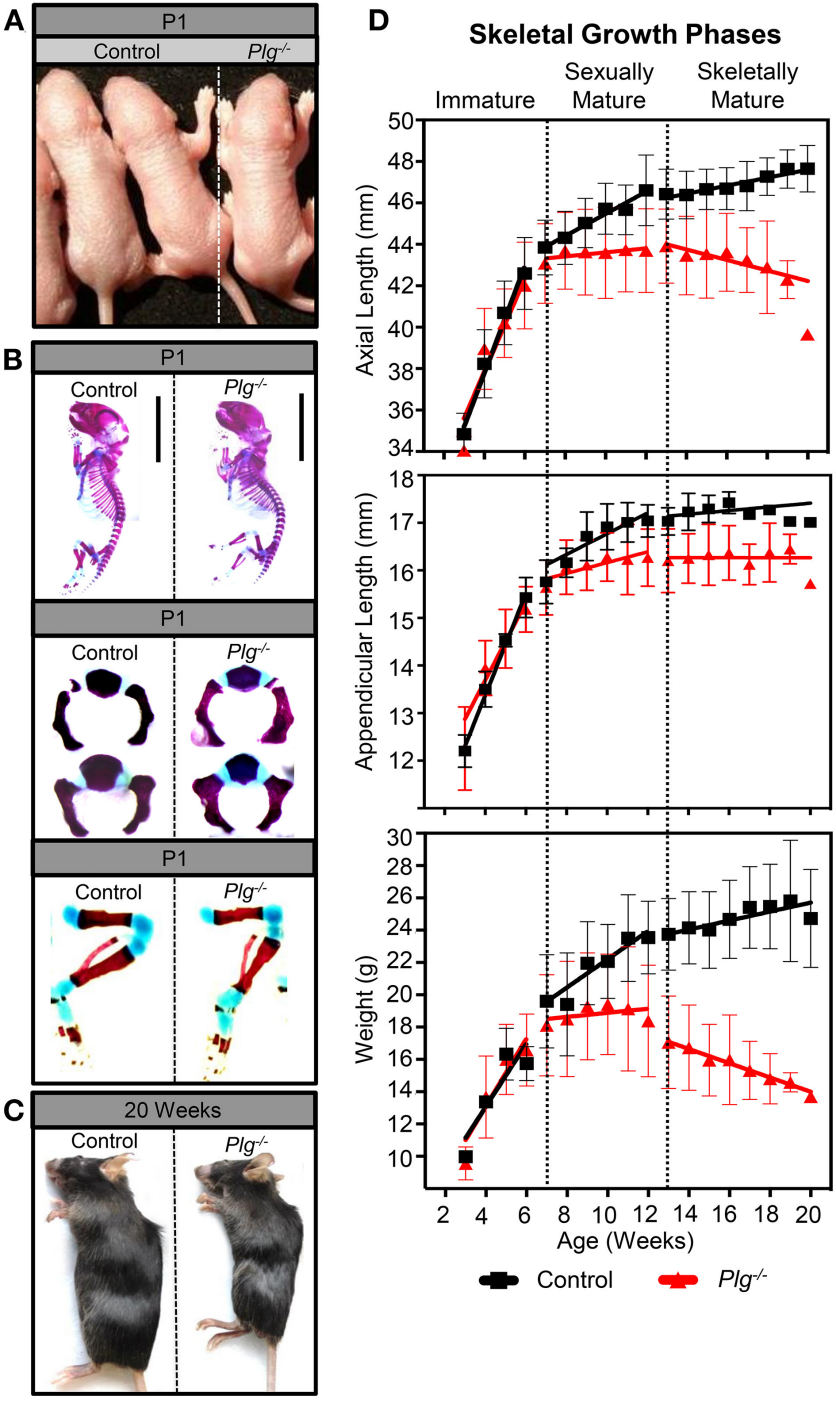
Plasminogen deficiency does not affect prenatal growth or skeletal development. At postnatal day 1 (P1), **(A)** gross examination and **(B)** alcian blue/alizarin red staining (blue indicates cartilage; red indicates mineralized bone) of control (wild-type littermates, WT) and *Plg*^−/−^ male mice demonstrate no observable differences in prenatal growth and skeletal development. However, after sexual and skeletal maturity (20 weeks of age), **(C)** gross examination of *Plg*^−/−^ mice shows stunted growth compared to control littermates. **(D)** Serial measurements of axial and appendicular skeletal lengths (measured radiographically) and body weights of WT and *Plg*^−/−^ mice during immature (3–6 weeks of age), sexually mature (7–12 weeks of age), and skeletally mature (13–20 weeks of age) growth phases. Error bars represent SD. See Supplementary Table 1 for number of mice quantified and Supplementary Table 2 for statistical analyses of growth rates. Statistical differences between WT and *Plg*^−/−^ mice growth rates were examined by the use of analysis for covariance (ANCOVA).

Gross and histologic analysis of mice with reduced fibrinolytic potential due to a genetic loss of plasminogen (*Plg*^−/−^) revealed that a loss of fetal plasminogen does not detectably affect skeletal development *in utero* (Figures 1A,B). Alcian blue/alizarin red staining of WT and *Plg*^−/−^ skeletons at P1 showed no obvious differences in prenatal development of the chondroepiphyses (blue) and primary ossification centers (red) within the axial or appendicular skeleton (Figure 1B). Alternatively, gross examination at 20 weeks of age revealed a wasting phenotype in plasminogen deficient mice as described previously (16–18), with markedly diminished animal length and weight compared to WT littermate control (Figure 1C; Supplementary Figure 2).

While we observed no differences in body weight, appendicular length, or axial length between WT mice and those with deficient fibrinolytic potential (*Plg*^−/−^) before sexual maturity (immature growth phase; 3–6 weeks; Figure 1D), *Plg*^−/−^ mice underwent a sharp decline in axial growth rate after sexual maturity, but before skeletal maturity (sexually mature growth phase; 7–12 weeks), coinciding with a reduced rate of appendicular length and weight gain. Furthermore, after skeletal maturity (13–20 weeks), early growth cessation was denoted in mice with plasminogen deficiency indicated by significant shortening of the axial and appendicular skeletons, weight loss, and subsequent kyphosis (Figure 1; Supplementary Table 2).

### Impaired Fibrinolytic Potential Results in Physeal Abnormalities and Decreased Vasculature in the Distal Femur

**Figure 2:**
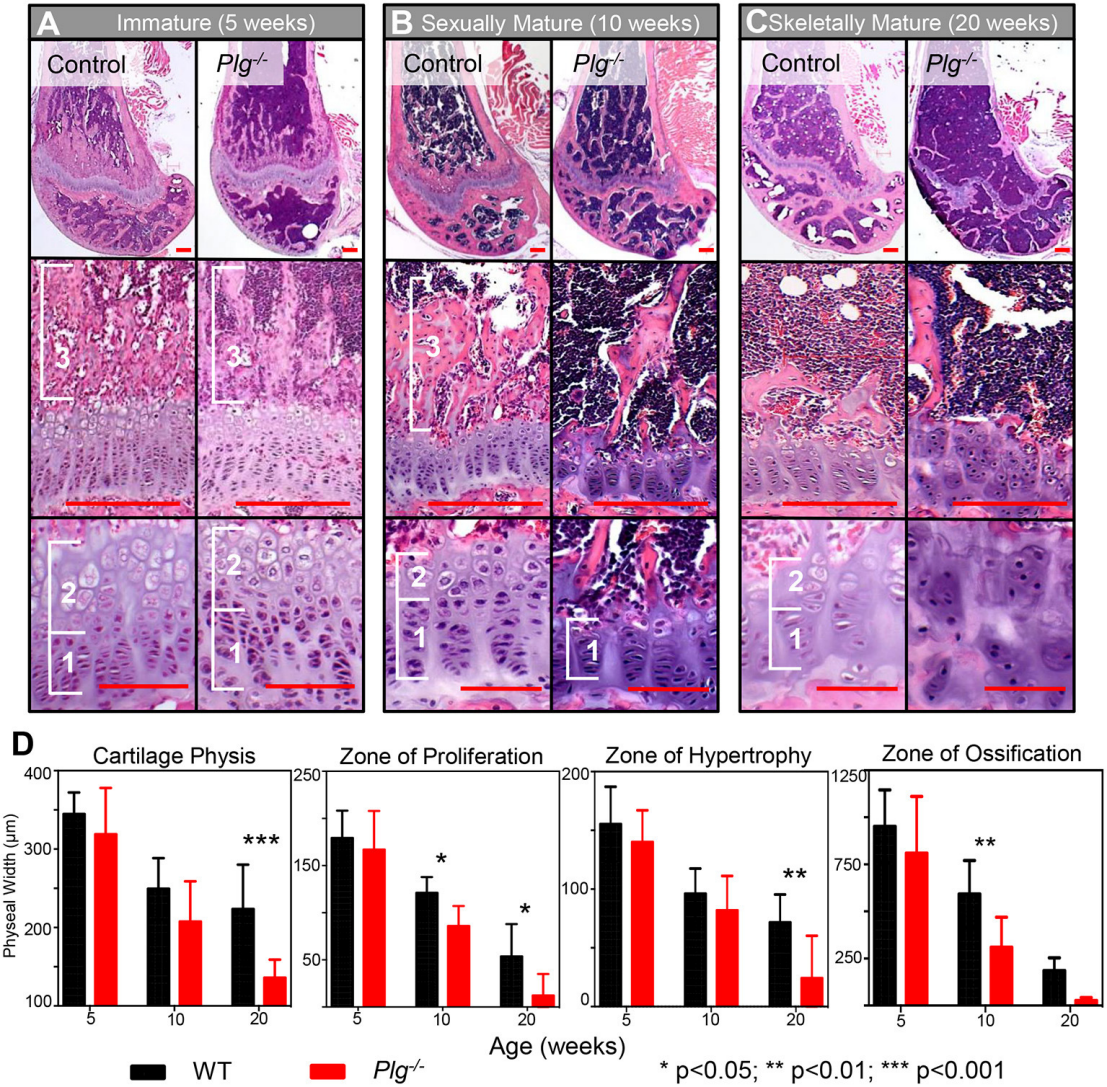
Impaired fibrinolysis, as a result of a plasminogen deficiency, results in postnatal physeal abnormalities and early cessation of skeletal growth. Histological evaluation of the distal femur of *Plg*^−/−^ and WT mice during **(A)** immature (5 weeks), **(B)** sexually mature (10 weeks), and **(C)** skeletally mature (20 weeks) growth phases. Physeal zones are demarcated with white brackets (1, zone of proliferative; 2, zone hypertrophy; and 3, zone of ossification). No apparent differences in zone sizes or organization were observed in WT and *Plg*^−/−^ mice during immature skeletal development. In contrast, *Plg*^−/−^ mice in the sexually mature and skeletally mature growth phases showed reduced widths of the proliferative and hypertrophic zones in comparison to WT age-matched mice. Additionally, the physis of *Plg*^−/−^ mice during both of these growth phases was markedly disorganized, indicating premature cessation of growth [H&E, Top Panel: 2.5× magnification (red scale bars, 100 μm); Middle Panel: 20× magnification (red scale bars, 25 μm); Bottom Panel: 40× magnification (red scale bars, 100 μm)]. **(D)** Quantification of physeal zones in wild type and *Plg*^−/−^ mice during skeletal development. Measurement of cartilaginous physis, zone of hypertrophy, zone of proliferation, and zone of ossification at immature (5 weeks), sexually mature (10 weeks), and skeletally mature (20 weeks) growth phases in *Plg*^−/−^ and WT mice (2-way ANOVA; reported *P*-values are from *post-ho*c Sidak corrections comparing *Plg*^−/−^ vs. WT mice). *p < 0.05; **p < 0.01; ***p < 0.001.

Given the observed changed in skeletal growth, histologic analysis of the distal femur during the different growth phases was examined in WT and *Plg*^−/−^ mice (Figure 2). During the immature growth phase (3–6 weeks of age), analysis revealed normal development and organization of the physis in *Plg*^−/−^ mice as compared to WT (Figure 2A). However, during sexual maturity (7–12 weeks of age), the widths of the physeal proliferative and ossification zones were markedly reduced in *Plg*^−/−^ mice compared to WT (Figures 2B,D). After skeletal maturity (20 weeks), we observed marked disorganization on the physis in *Plg*^−/−^, evident by the reduction of detectable physeal zones or tidemarks (Figures 2C,D). Notably, the physes in both groups of mice reached senescence at skeletal maturity, as demonstrated by the plateau in growth rates of the appendicular skeleton. Yet, the disorganized and reduced zones of proliferation, hypertrophy, and ossification seen at skeletal maturity in *Plg*^−/−^ mice may be indicative of a reduction in the proliferative capacity of physeal chondrocytes and corresponded to the significant decline in appendicular growth rate observed during this growth phase.

**Figure 3:**
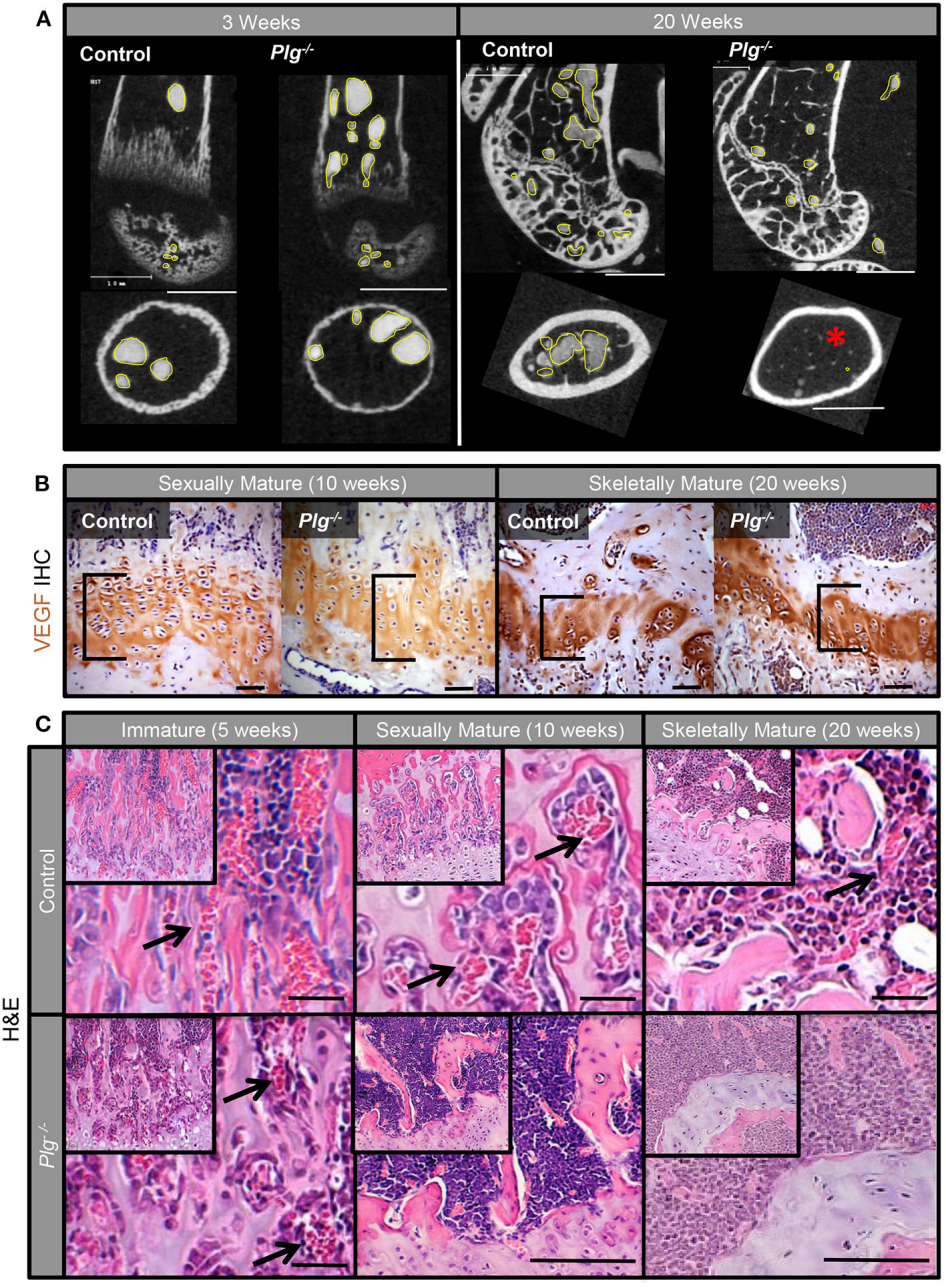
Impaired fibrinolysis, as a result of a plasminogen deficiency, leads to the loss of acquired physeal vasculature. **(A)** Angiography of the distal femur of *Plg*^−/−^ and WT control mice at 3 weeks of age demonstrated that *Plg*^−/−^ mice develop a normal epimetaphyseal vascular supply. After skeletal maturity (20 weeks of age), there is markedly reduced epimetaphyseal and epiphyseal vasculature in *Plg*^−/−^ mice compared to WT controls [2D μCT images; vascular spaces outlined in yellow; red asterisk denotes lack of metaphyseal vessels (white scale bars, 1 mm)]. **(B)** Immunohistochemical staining for vascular endothelial growth factor (VEGF) shows no discernable differences in VEGF immunoreactivity within the physis (black brackets) of WT control and *Plg*^−/−^ mice at 10 and 20 weeks of age [magnification 40× (black scale bars, 20 μm)]. **(C)** H&E stains of the physis in *Plg*^−/−^ and WT control mice at 5, 10, and 20 weeks of age. Whereas, the physeal vasculature (black arrows) is comparable in *Plg*^−/−^ and WT mice at 5 weeks of age (immature phase), the physeal vasculature within the zone of provisional ossification is markedly reduced in *Plg*^−/−^ mice at 10 and 20 weeks of age [H&E, magnification 20× inset; 40× main image (black scale bars, 100 μm)].

In addition to changes in physeal architecture, angiography of the distal femur also demonstrated progressive loss of microvasculature in the distal metaphysis and epiphysis of mice with reduced fibrinolysis (*Plg*^−/−^) (Figure 3A). While abundant metaphyseal vasculature was apparent in vascular casts of both WT and *Plg*^−/−^ mice at 3 weeks of age, vascularity diminished in *Plg*^−/−^ mice by skeletal maturity (20 weeks) (Figure 3A). Yet, immunohistochemical staining for vascular endothelial growth factor (VEGF), a potent angiogenic stimulator, revealed no detectable differences in VEGF protein levels within the physis of WT and *Plg*^−/−^ mice at 10 or 20 weeks of age (Figure 3B). Therefore, the dramatic change in the number of blood vessels supplying the zone of ossification in *Plg*^−/−^ mice (Figures 3A,C) is not a result of reduced, local VEGF production or accumulation.

### The Proximal Femur Fails to Develop Normally in Mice With Impaired Fibrinolysis

**Figure 4:**
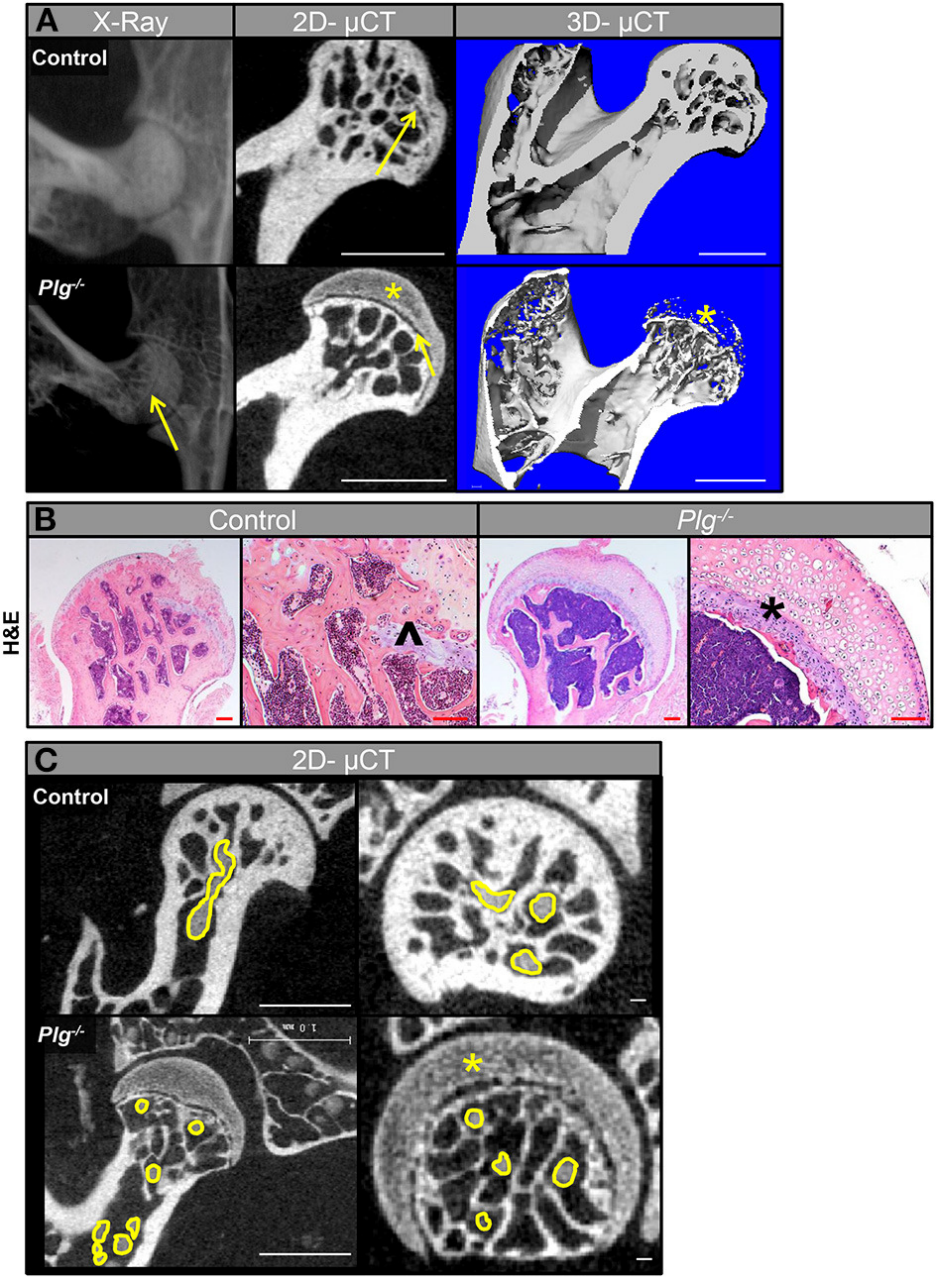
Vascularization and subsequent ossification of the proximal femoral epiphysis in mice with impaired fibrinolysis. **(A)** Radiographs (*in vivo*) and μCT images showed that the proximal epiphysis is ossified with partial physeal closure (yellow arrow) in WT control mice at 13 weeks of age, but that the proximal epiphysis remains unossified (yellow asterisk) and the physis remains open (yellow arrow) in *Plg*^−/−^ littermates (scale bars, 1 mm). **(B)** Histological analysis of femoral heads at 13 weeks of age confirmed ossification of the epiphysis and vascular penetration beyond the physeal remnant (black caret) in the control mice, whereas the epiphysis in *Plg*^−/−^ mice is composed of disorganized hypertrophic cartilage (black asterisk) and an inactive physis [H&E, magnification 2.5× and 10× (scale bars, 100 μm)]. **(C)** 2D-μCT images from mice injected with angiogram contrast material at 20 weeks of age delineated the epiphyseal vascular supply of the proximal femur in WT control and *Plg*^−/−^ mice (vasculature outlined in yellow). 2D-μCT images also show that control mice have an ossified epiphysis and a fully resorbed physis by 15 weeks of age, whereas *Plg*^−/−^ mice have not ossified the epiphysis (yellow asterisk) and instead have open physes (left panel: scale bars, 1 mm; right panel: scale bars, 100 μm).

Radiographic and μCT analyses of WT mice confirmed that physeal resorption and epiphyseal ossification began at 13 weeks in WT mice (skeletally mature growth phase) as previously described (12). Alternatively, in *Plg*^−/−^, the proximal epiphysis remained unossified, with a complete and open physis (Figure 4A). To corroborate these findings, the femoral heads from mice at 13 weeks of age were examined histologically. WT mice showed ossification of the epiphysis with vascular penetration beyond the physeal remnant, whereas the epiphysis in *Plg*^−/−^ mice remained unossified and was composed of hypertrophic, disorganized cartilage with an inactive physeal plate (Figure 4B). Aligning with prior observation at the distal femur, *Plg*^–/–^ mice likewise failed to develop vascular invasion across the physis to the cartilaginous anlage of the proximal femur, thus precluding ossification of the proximal femoral epiphysis (Figure 4C; Table 1).

**Table 1:** Quantification of the ossification of the proximal femoral epiphysis in mice with impaired fibrinolysis.

**Genotype**	**Number of ossified epiphyses (10 weeks)**	**Bone mineral density (mgHA/ccm) (10 weeks)**	**Number of ossified epiphyses (20 weeks)**	**Bone mineral density (mgHA/ccm) (20 weeks)**
Control	0/5	616.84 ± 38.96	5/5	894.49 ± 44.77
*Plg^−/−^*	0/12	591.25 ± 7.72	0/8	650.20 ± 35.43^*^

* *Denotes p < 0.05 as compared to control*.

*Number of ossified epiphyses and bone mineral density in WT and Plg^−/−^ sexually mature (10 weeks) and skeletally mature mice (20 weeks). At 20 weeks, all of the WT control mice showed epiphyseal ossification. In contrast, none of the Plg^−/−^ mice showed ossified epiphyses. In addition, differences in bone mineral density between Plg^−/−^ and WT control mice were significant at 20 weeks of age (Fisher's exact test, p = 0.036)*.

### Bone Formation Perimeters Are Altered in Mice With Impaired Fibrinolysis

**Figure 5:**
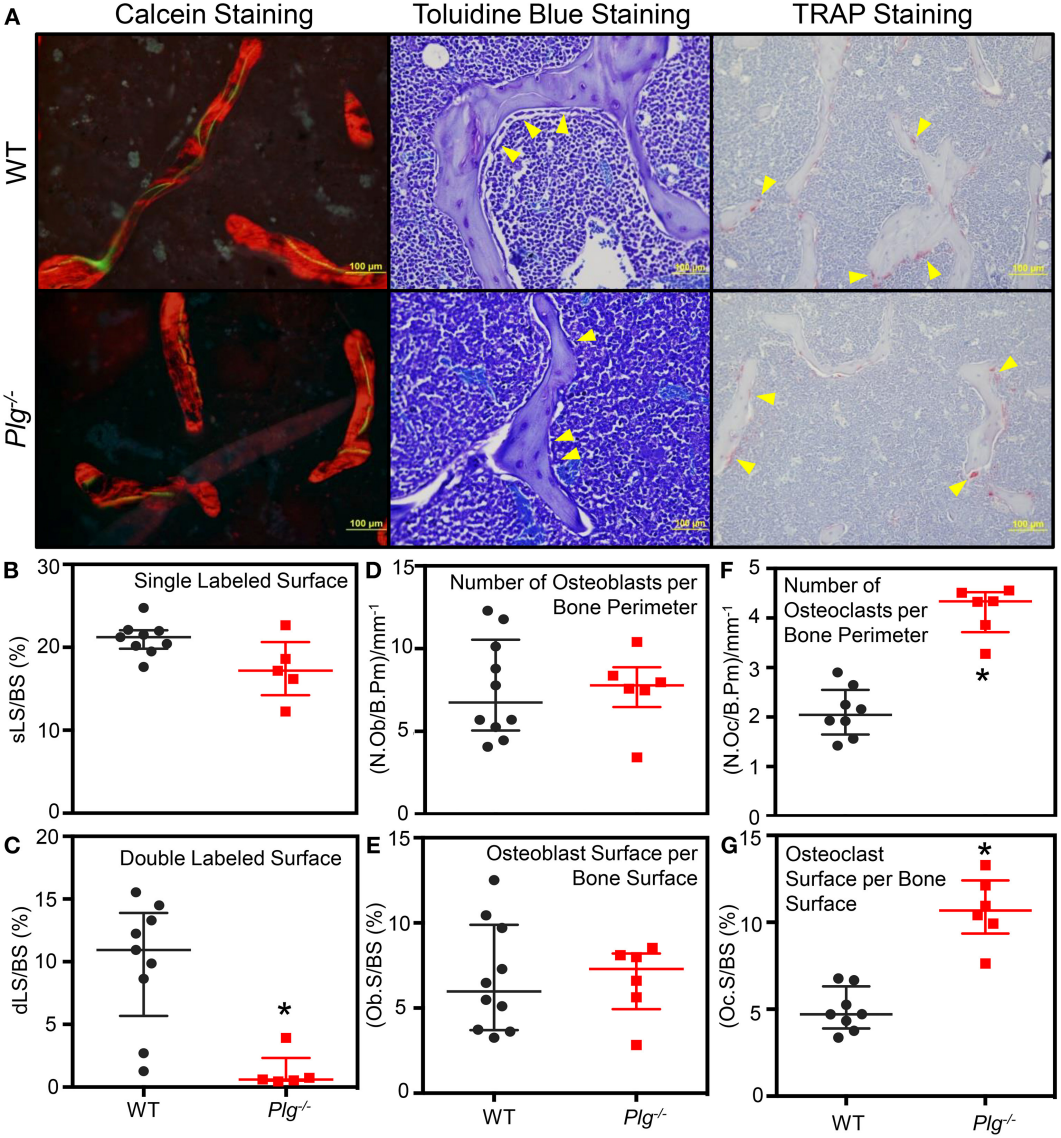
Impaired fibrinolysis, as a result of a plasminogen deficiency, leads to impaired bone remodeling and bone volume. **(A)** Representative images from the histological analysis of the secondary spongiosa from the distal femur of *Plg*^−/−^ and WT control mice at 10 weeks of age by calcein (20×), Toluidine blue (40×), and TRAP staining (20×). *Plg*^−/−^*N* = 3, WT control *N* = 4 (Calcein Staining) and WT *N* = 5 (Toluidine blue and TRAP staining), two sections analyzed per mouse. **(B–G)** Bone histomorphometic analysis of the secondary spongiosa of the distal femur of *Plg*^−/−^ and WT control mice at 10 weeks of age. The percent of single labeled surface per bone surface (sLS/BS) **(B)** and the percent of double labeled surface per bone surface (dLS/BS) **(C)** were analyzed following calcein injections at 10 and 3 days prior to sacrifice. The number of osteoblasts per bone parimeter (N.Ob/B.Pm) **(D)** and the percentage of osteoblast surface per bone surface (Ob.S/BS) **(E)** were analyzed following Toluidine blue staining. The number of osteoclasts per bone perimeter (N.Oc/B.Pm) **(F)** and the percentage of osteoclast surface per bone surface (Oc.S/BS) **(G)** were analyzed following TRAP staining. All analyses were statistically evaluated using unpaired *t*-test with multiple comparisons and a false discovery rate of 5%. Please see Supplementary Table 3 for an extended analysis of histomorphometry parameters. *p < 0.05.

To analyze the bone elements in more detail, histomorphometric analysis of WT and *Plg*^−/−^ mice at 10 weeks of age was conducted (Figure 5; Supplementary Table 3). Calcein staining demonstrated a marked decrease in trabecular number, trabecular thickness, bone volume (BV) [as a measure of Bone volume/tissue volume (BV/TV)], as previously reported (12), as well as a decreased percent mineralized surface (MS) per bone surface (BS): MS/BS in mice with impaired fibrinolysis compared to WT controls (Supplementary Table 3). Furthermore, we observed significantly reduced double label staining (Figures 5A,C), but comparable single label staining (Figure 5B) in *Plg*^−/−^ mice as compared to WT controls, indicating altered bone formation. Analysis of osteoblast count by morphology revealed reduced osteoblast numbers in *Plg*^−/−^ mice compared to WT controls (Supplementary Table 3), yet comparable numbers per bone parameter (N.Ob/B.Pm) and osteoblast surface per bone surface (Ob.S/BS) (Figures 5A,D,E). Analysis of osteoclasts *via* TRAP staining revealed comparable osteoclast numbers (Supplementary Table 3), yet significantly greater number of osteoclasts per bone perimeter (N.Oc/B.Pm) and osteoclast surface per bone surface (Oc.S/BS), aligning with the reduced trabecular bone volume experience in *Plg*^−/−^ mice (Figures 5A,F,G). Taken together these findings further indicate an impairment of ossification at the physis and suggest potential uncoupling of the osteoblast and osteoclast unit in mice with impaired fibrinolysis (*Plg*^−/−^).

### Genetically Reducing IL-6 Levels Does Not Rescue Skeletal Growth in Mice With Impaired Fibrinolysis

Previous work has demonstrated that persistent fibrin deposition induces production of IL-6, a potent stimulator of inflammatory responses, which in turn further stimulates fibrinogen production in the liver leading to a chronic inflammatory state (10, 19). Given that chronic inflammation has been linked clinically with skeletal and growth abnormalities in children, we examined if reduction of IL-6 is sufficient to rescue the skeletal abnormalities observed in *Plg*^−/−^ mice.

**Figure 6:**
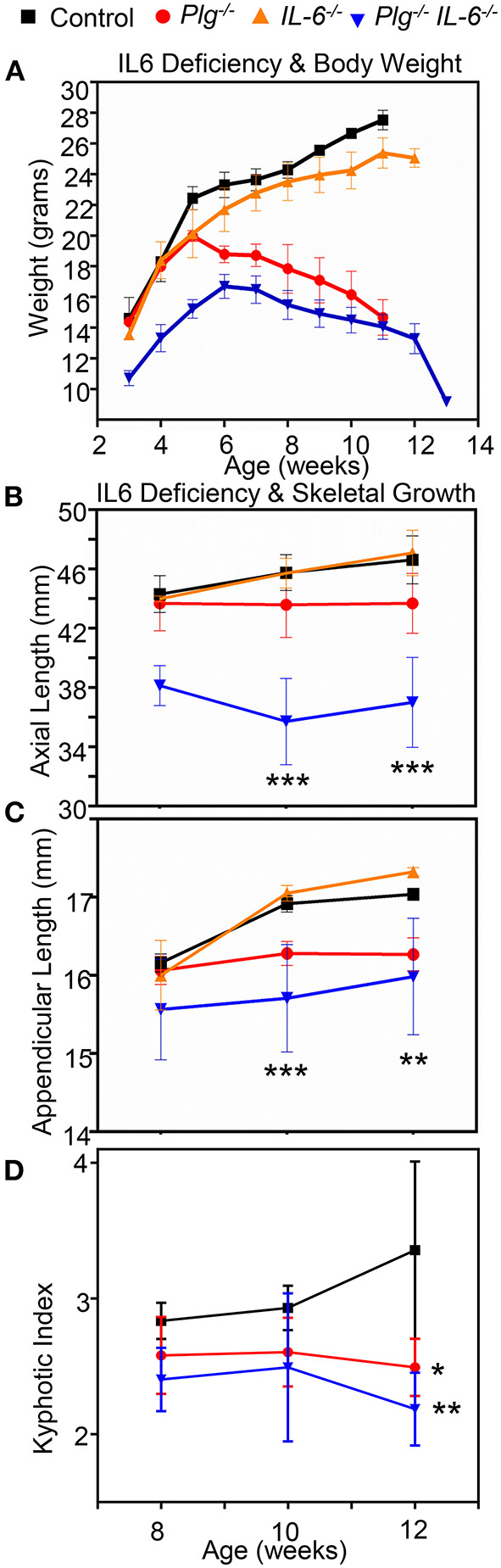
Reduction of interleukin-6 does not rescues the skeletal growth phenotypes seen in mice with impaired fibrinolysis. **(A)** Comparison of *Plg*^−/^ *IL6*^−/−^*, Plg*^−/−^*, IL6*^−/−^ mice and WT littermate controls weight curve from 3 to 13 weeks shows a significant difference in the *Plg*^−\−^ *IL6*^−\−^ compared to control. **(B)** Axial length as measured by radiographic tibial analysis shows significant differences at 10 and 12 weeks of *IL6*^−/−^ *Plg*^−/−^ compared to control wildtype mice. **(C)** Appendicular length as measured by radiographic vertebral analysis demonstrates significant differences in *IL6*^−/−^ *Plg*^−/−^compared to control mice at 10, and 12 weeks. **(D)** Kyphotic index as measured by radiographic analysis was likewise significantly different in both *Plg*^−/−^ and *IL*6^−/−^ mice compared to control mice at 12 weeks of age. ***p < 0.001, **p < 0.01, and *p < 0.05 respectively. Error bars represent SD. See Supplementary Table 2 for quantification and statistical analysis.

Contrary to our hypothesis, when *Plg*^−/−^ mice were crossed with those deficient in IL-6 (*Plg*^−/−^ *IL-6*^−/−^*)*, we did not observe any improvement in skeletal pathology (Figure 6). While IL-6 deficient mice (*IL-6*^−/−^) were comparable to WT controls for all parameters assessed, *Plg*^−/−^ *IL-6*^−/−^ mice actually possessed a further reduction in appendicular length, axial length, body weight, and worsened kyphotic index beyond that of *Plg*^−/−^ mice alone (Figure 6).

### Genetically Reducing Fibrinogen Levels Rescues Skeletal Growth and Systemic Vascularity in Mice With Impaired Fibrinolysis

**Figure 7:**
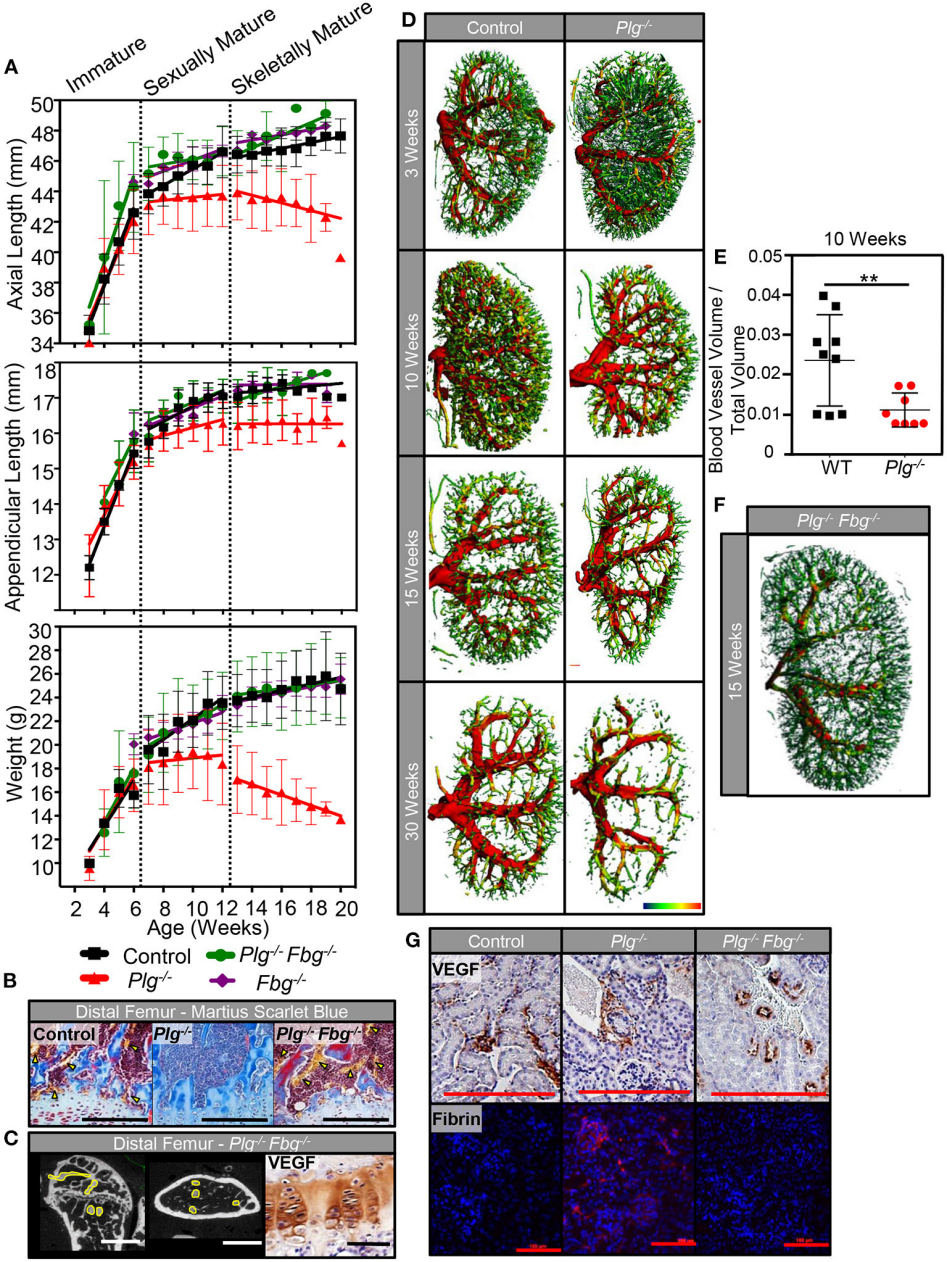
Genetic reduction of fibrin(ogen) rescues skeletal development and reduction in systemic vascularity associated with impaired fibrinolysis. **(A)** Serial body weight, axial, and appendicular skeletal length (measured radiographically) of WT control, *Plg*^−/−^, and *Plg*^−/−^ *Fbg*^−/−^ mice during immature, sexually mature, and skeletally mature growth phases. **(B)** Histological examination of the distal femurs from WT control, *Plg*^−/−^, and *Plg*^−/−^ *Fbg*^−/−^ mice at 20 weeks of age demonstrated preservation of the metaphyseal blood supply in *Plg*^−/−^ *Fbg*^−/−^ mice, compared to the complete absence of vasculature in *Plg*^−/−^ mice [Martius scarlet blue stain; erythrocytes are stained yellow and physeal vessels are indicated with yellow arrowheads (10×, scale bars, 100 μm)]. **(C)** Angiography of distal femurs shows an intact metadiaphyseal vasculature in *Plg*^−/−^ *Fbg*^−/−^ mice at 20 weeks of age [2D μCT, vasculature outlined in yellow (scale bars, 1 mm)]. There was no detectable change in VEGF immunoreactivity seen in the distal physes of *Plg*^−/−^ *Fbg*^−/−^ mice as measured by immunohistochemical staining (40×, scale bars, 25 μm). **(D)** Kidneys from *Plg*^−/−^ mice and WT control littermates were analyzed by 3D μCT angiography at 3, 10, 15, and 30 weeks of age for renal vascularity. While both control and *Plg*^−/−^ kidneys undergo a reduction in renal vascularity with age, *Plg*^−/−^ mice appear to lose renal microvasculature at an accelerated rate beginning at 10 weeks of age. **(E)** Quantification of blood vessel volume and total tissue volume obtained from μCT following angiography of *Plg*^−/−^ mice and control littermates at 10 weeks of age (**p < 0.01). **(F)** Kidneys from *Plg*^−/−^ *Fbg*^−/−^ at 15 weeks of age (following skeletal maturity) were analyzed by 3D μCT angiography to demonstrate rescue of the renal vasculature when fibrin(ogen) is genetically reduced in the setting of plasminogen deficiency. **(G)** Immunohistochemical staining for VEGF in the glomeruli of *Plg*^−/−^, and *Plg*^−/−^ *Fbg*^−/−^, and WT control littermates following skeletal maturity (20×, scale bars, 100 μm). Immunolocalization of fibrin within glomeruli of *Plg*^−/−^, *Plg*^−/−^ *Fbg*^−/−^, and WT control littermates following skeletal maturity (10×, scale bars, 100 μm).

While genetic reduction in IL-6 did not improve skeletal growth in mice with impaired fibrinolysis, genetic reduction of fibrinogen levels (*Plg*^−/−^ *Fbg*^−/−^) completely rescued the abnormalities in skeletal growth seen in *Plg*^−/−^ mice (Figure 7). Unlike *Plg*^−/−^ mice, *Plg*^−/−^ *Fbg*^−/−^ mice showed no marked differences in body weight, axial length, or appendicular length compared to WT control mice (Figure 7A; Supplementary Table 2). Likewise, mice deficient in fibrinogen alone (*Fbg*^−/−^) did not show significant differences in body weight, axial length, or appendicular length compared to WT control mice. Taken together, these findings indicate that the skeletal changes in *Plg*^−/−^ mice are potentially linked to fibrin deposition, independent of an IL-6 mediated hyperinflammatory environment.

In addition to improvement in skeletal growth, vascular analysis of *Plg*^−/−^ *Fbg*^−/−^ mice showed restoration of the vascular supply at the zone of ossification in the distal femur (Figure 7B). This finding was also supported by angiographic analysis (Figure 7C), which showed a vascular pattern comparable to WT controls (Figure 3A). Furthermore, akin to results shown in Figure 3B, immunohistochemical staining for VEGF revealed no marked differences in VEGF protein levels at the physis of the distal femur in *Plg*^−/−^*Fbg*^−/−^ and WT mice (Figure 7C).

To determine whether blunted vascular anatomy in *Plg*^−/−^ mice was specific to the bone/physes or was instead a systemic finding, 3D μCT reconstructions of renal vascular patterns were analyzed during all three growth phases to evaluate the effect of impaired fibrinolysis on an independent vascular network (Figure 7D). While no differences were observed in the renal vasculature at 3 weeks of age, *Plg*^−/−^ mice had comparably less renal vasculature than WT mice starting at 10 weeks of age (Figures 7D,E) and continuing throughout skeletal maturity. Prior reports have suggested that a baseline, when uninjured or un-challenged, *Plg*^−/−^ and WT animals have comparable renal function (20, 21). Taken together with this study, this suggests that the impaired skeletal development observed in *Plg*^−/−^ mice is likely not secondary to renal dysfunction and a downstream metabolic bone disease such as renal osteodystrophy.

Aligning with findings in the physis, when fibrinogen was genetically eliminated (*Plg*–/–*Fbg*^−/–^), renal vasculature was likewise rescued (Figure 7F), demonstrating that impaired fibrinolysis not only impacts skeletal growth and vascular integrity in the developing skeleton, but also systemically in other vital organ systems. Furthermore, immunohistochemical staining for VEGF failed to disclose marked changes in the number of vessels or VEGF protein levels in the kidney of *Plg*^−/−^, *Plg*^−/−^ *Fbg*^−/−^, or WT mice, confirming that the effect of impaired fibrinolysis on vascularity are independent of changes in VEGF protein levels (Figure 7G). In contrast, immunolocalization of fibrin showed considerable deposition within kidneys of *Plg*^−/−^ mice, but not in WT or *Plg*^−/−^ *Fbg*^−/−^ mice, implicating the inability to clear persistent fibrin deposition as an etiological contributor to the observed vascular and skeletal growth phenotypes (Figure 7G).

## Discussion

Here we show that mice deficient in plasminogen, the principal protease of removing fibrin, have hallmarks of premature skeletal aging and stunted growth. This study demonstrated that persistent fibrin deposition, but not elevated IL-6, is the mediator of early skeletal aging and physeal disruption in these mice that mimic chronic inflammatory conditions. These effects on skeletal development are associated with diminished microvascular networks, either as a result of reduced patency or failed vasculaogenesis. Our data supports the concept that the inability to clear fibrin deposits is a predominant etiological determinant for vascular regression and decreased osteogenic capacity during skeletal development. Thus, we conclude that adequate fibrinolytic activity is required to prevent premature skeletal aging and stunted growth by promoting the maintenance and development of the skeletal vasculature.

Fibrinogen has been shown to increase blood viscosity, vascular reactivity, and decreased endothelial integrity (22–29). Therefore, existing literature strongly supports hyperfibrinogenemia as a potential mediator of vascular dysfunction (26). As chronic inflammatory conditions in children with skeletal disturbances have been shown to have elevated fibrinogen, these skeletal effects may be due to deleterious effects in the microcirculation. In accordance with this hypothesis, our findings suggest that persistent fibrin deposition is associated with abnormal growth phenotypes as a result of dysregulated microvasculature of both the epiphysis and metaphysis. These findings align with clinical observations of physeal changes as a result of vascular insult (30–32). Through detailed histologic and angiogram analysis, we observed rarefaction of the epiphyseal blood supply that correlated to regression of proliferating chondrocytes, reduced terminal differentiation of hypertrophic chondrocytes, and subsequently impaired ossification. Importantly, following genetic elimination of fibrinogen, we observed correction of the vascular and developmental phenotypes. Thus, while there is a clear link between fibrinolytic activity and vasculogenesis, the specific mechanism is still unknown.

Importantly, these present studies cannot delineate if the observed phenotype is a result of deposited fibrin or fibrinogen. Future studies employing novel murine lines such as the FbgAEK mouse, where fibrinogen levels are unchanged but fibrin formation is impaired, would help determine if the abnormal growth phenotype observed in plasminogen deficient animals is due to persistent fibrin and/or fibrinogen. Aligning with our overarching hypothesis that fibrin deposition drives skeletal aging due to impaired fibrinolysis, we anticipate that FbgAEK crossed with plasminogen deficient mice will result in a rescue of the observed phenotype. Likewise, we anticipate that targeted inhibition of thrombin may also improve the observed phenotype in plasminogen deficient mice, by reducing the amount of fibrin formation during skeletal development. These two experimental conditions are to be evaluated in future studies.

Given that IL-6 is the principle initiating cytokine in the acute phase response (33, 34) and thought to be the principle driver of fibrinogen overexpression, we hypothesized that IL-6 would contribute both directly to the observed pathology through IL-6 receptors on inflammatory cells (including inducing osteoclastogenesis) and indirectly through promotion of fibrinogen production; thereby exacerbating its pathologic inflammatory and vascular effects. Surprisingly, we found that IL-6 was negligible to the pathology observed in mice with impaired fibrinolysis. Rather, *Plg*^−/−^ *IL6*^−/−^ mice actually demonstrated a greater reduction in weight, bone length, and bone volume following sexual maturity compared to *Plg*^−/−^ mice alone. We hypothesize that this may be due to alternative inflammatory pathways compensating following the genetic loss of IL-6, such as TNF-a (35). However, these results do clearly demonstrate that the IL-6-mediated inflammatory cascade does not drive the observed bone and vascular pathologies, and thus, modulation of IL-6 does not represent a viable therapeutic option to mediating inflammatory skeletal conditions associated with impaired fibrinolysis. Given the strong associations clinically between chronic inflammation and skeletal defects, investigation of alternative inflammatory pathways that are regulated by fibrin deposition and can drive the observed bone and vascular phenotypes, is warranted. We hypothesize that such pathways include IL-1/IL-β, which has been demonstrated to regulate osteoclast differentiation and bone resorption, in addition to being positively modulated by the presence of fibrinogen (36, 37). Given the variety of IL-1 targeted therapies available and their successful utilization for treatment of rheumatoid arthritis, expanded investigations of IL-1 in the context of inflammatory skeletal conditions with persistent fibrinogen deposition are warranted.

In conclusion, persistent fibrin deposition as a result of impaired fibrinolysis from a plasminogen deficiency, leads to premature skeletal aging and stunted growth, independent of IL-6. Diminished vascularity and/or chronic inflammation are key drivers of impaired skeletal development. These findings demonstrate that fibrin is an upstream culprit of these two observed pathologies in the plasminogen deficient animals. If these findings translate to other disease states, such as starvation, obesity, infection, chemotherapy, and autoimmune diseases, this would suggest that one potential therapeutic option would be to target either the deposition of fibrin and or enhancing fibrinolysis to restore the vascular deficiency and chronic inflammation which would in turn restore skeletal development.

## Data Availability Statement

The raw data supporting the conclusions of this article will be made available by the authors, without undue reservation.

## Ethics Statement

The animal study was reviewed and approved by Vanderbilt University IACUC.

## Author Contributions

HC, MY, LG, JN, MF, and JS contributed to conception and design of the study. HC, SM-L, GH, RJ, and MY contributed to data collection and analysis. HC, SM-L, and RJ contributed to figure production. HC, SM-L, and JS wrote the draft of the manuscript. SM-L, GH, LG, JN, MF, and JS contributed to manuscript preparation and review. JS obtained funding for the study. All authors contributed to manuscript revision, read, and approved the submitted version.

## Funding

This work was supported by the National Institutes of Health [(1R01GM126062-01A1, NIGMS, JS), (T32GM007628, NIGMS, SM-L)], the Vanderbilt University Medical Center Department of Orthopaedics and Rehabilitation (JS), the Jeffrey W. Mast Chair in Orthopaedics Trauma and Hip Surgery (JS), and the Caitlin Lovejoy Fund (JS). Use of the Translational Pathology Shared Resource was supported by NCI/NIH Cancer Center Support Grant (2P30 CA068485-14) and the Vanderbilt Mouse Metabolic Phenotyping Center Grant (5U24DK059637-13). μCT imaging and analysis were supported in part by the Center for Small Animal Imaging at the Vanderbilt University Institute of Imaging Sciences (S10RR027631) from the NIH. Grant 1S10OD021804-01A1 supported the Replacement and Upgrade of an Optical Imaging System for Small Animals, housed in the Vanderbilt Center for Small Animal Imaging, and used in this proposal. Funding sources for this project had no involvement in study design, collection, and analysis of data, writing of the report, or decision in submitting this article for publication.

## Conflict of Interest

JS receives research support from OrthoPediatrics, IONIS Pharmaceuticals, PXE International, the United States Department of Defense, and the National Institutes of Health. The remaining authors declare that the research was conducted in the absence of any commercial or financial relationships that could be construed as a potential conflict of interest.

## Publisher's Note

All claims expressed in this article are solely those of the authors and do not necessarily represent those of their affiliated organizations, or those of the publisher, the editors and the reviewers. Any product that may be evaluated in this article, or claim that may be made by its manufacturer, is not guaranteed or endorsed by the publisher.

## Supplementary Material

Supplementary Figure 1 Validation of radiographic skeletal measurements. To accurately measure growth over time, radiographic methods were validated for **(A)** measurement of spinal length (axial measurement) and tibial length (appendicular measurement). Axial radiographs were obtained with mice in the lateral decubitus position (see insert) and then analyzed using the segmented line tool in ImageJ. Appendicular radiographs were obtained with mice in the prone position (see insert). **(B)** Appendicular lengths were obtained from radiographic images by measuring the distance from the apex of the tibial plateau to the distal plafond using the straight-line tool in ImageJ. **(C)** To validate radiographic skeletal measurements, mouse length from nose to anus (left panel) and tibial length (right panel) were measured manually using standard digital calipers. **(D)** Comparisons of manual (X-axis) and radiographic measurements (Y-axis) of the tibia showed strong correlation (*R*^2^ = 0.91). The right shift of the linear regression from the line of identity (red line) indicates that radiographic methods are systematically lower than manual readings. Since the slope of the linear regression line is not significantly different from 1.0 (1.02 ± 0.06; *p* = 0.66), radiographic measurements were considered a reliable indicator of differences in appendicular bone length. **(E,F)** Serial measurement of tibial length (*N* = 4) in weekly radiographs of WT C57BL/6 mice from 3 to 20 weeks of age demonstrated significant growth deceleration around the onset of puberty (7–8 weeks of age). **(G)** Measurements of the spinal length determined from lateral radiographs were plotted on the Y-axis against manual (caliper) measurements on the X-axis (red line, line of identity). Radiographic measurements were markedly shorter than those obtained from caliper-based methods because of the extraspinal length (skull and sacrum) included in caliper measurements. Thus, the slope of the linear regression line is significantly <1.0 (0.53 ± 0.04; *p* < 0.0001) and measurements of spinal length by radiographic techniques are systematically smaller than manually measured body lengths, particularly when measuring longer spines (and skulls and sacra). Nevertheless, both measurements were strongly correlated (*R*^2^ = 0.92) and therefore radiographic measurements were considered a reliable indicator of differences in axial skeletal length. **(H,I)** Serial spinal lengths in WT C57BL/6 mice (*N* = 4) measured in serial lateral radiographs from 3 to 20 weeks of age also demonstrated marked deceleration of growth rate around the onset of puberty (6–8 weeks of age).

Supplementary Figure 2 Differences in gross postnatal development of wild type and plasminogen-deficient mice. Gross examination of *Plg*^−/−^ mice and WT littermate controls demonstrated normal axial length and overall size during the immature growth phase (5 weeks of age), but stunted growth in sexually mature (10 weeks of age) and skeletally mature animals (20 weeks of age).

Supplementary Table 1 Number of mice quantified per skeletal development measure per week. WT, *Plg*^−/−^*, Fbg*^−/−^*, Plg*^−/−^*Fbg*^−/−^*, IL-6*^−/−^*, and Plg*^−/−^*IL-6*^−/−^ mice were analyzed for axial length, appendicular length, and weight each week following weaning at 3 weeks post-birth. The number of mice evaluated at each time point for each output is presented.

Supplementary Table 2 Quantification and analysis of growth curves mice at immature (5 weeks), sexually mature (10 weeks), and skeletally mature (20 weeks) growth phases. Statistical significance between genotypes was calculated by linear regression and analysis for covariance between groups (ANCOVA). Rate of Growth: Analysis of growth curves was conducted to investigate statistical differences between the slope of the linear regression across the immature, sexually mature, and skeletally mature growth phases. In cases where differences between slopes were non-significant, absolute measurements (and therefore the Y-intercepts) were analyzed. In cases where differences between slopes were significant, it was not possible to test whether the intercepts differed significantly. Statistical analysis was conducted on the rate of axial growth, appendicular growth, and weight gain.

Supplementary Table 3 Histomorphometic Analysis of the Secondary Spongiosa of Distal Femurs from 10-week-old male *Plg*^−/−^ and WT control mice. Values represent mean ± SD. All analyses were statistically evaluated using unpaired *t*-tests with multiple comparisons and a false discovery rate of 5%.
