# Supplementary figures and images for: The Deleterious Effects of Impaired Fibrinolysis on Skeletal Development Are Dependent on Fibrin(ogen), but Independent of Interlukin-6
Source: Front Cardiovasc Med. 2021 Dec 6;8:768338. doi: 10.3389/fcvm.2021.768338 (PMC8685342; doi:10.3389/fcvm.2021.768338)

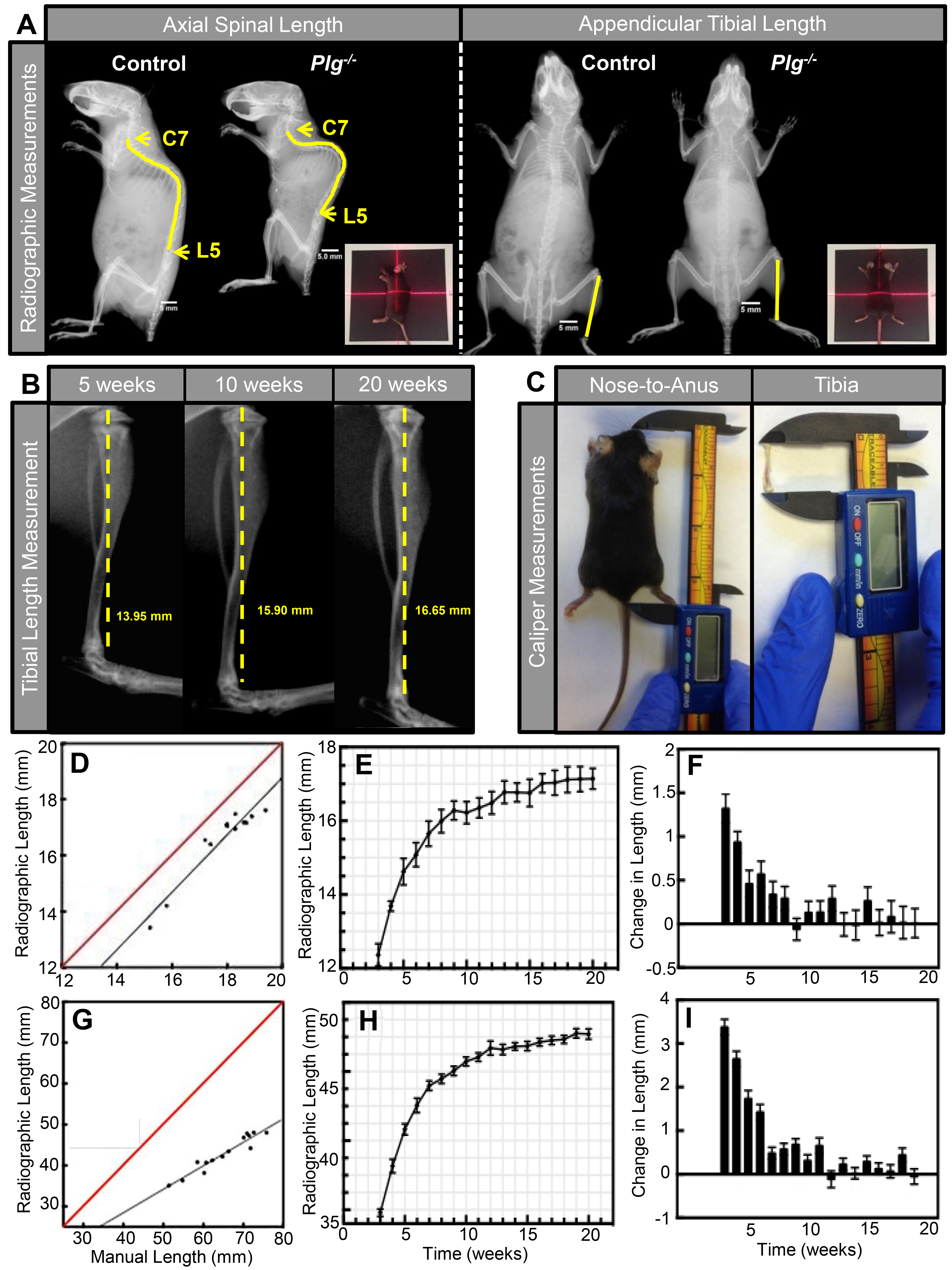

Supplement: Supplementary Figure 1 — Validation of radiographic skeletal measurements. To accurately measure growth over time, radiographic methods were validated for (A) measurement of spinal length (axial measurement) and tibial length (appendicular measurement). Axial radiographs were obtained with mice in the lateral decubitus position (see insert) and then analyzed using the segmented line tool in ImageJ. Appendicular radiographs were obtained with mice in the prone position (see insert). (B) Appendicular lengths were obtained from radiographic images by measuring the distance from the apex of the tibial plateau to the distal plafond using the straight-line tool in ImageJ. (C) To validate radiographic skeletal measurements, mouse length from nose to anus (left panel) and tibial length (right panel) were measured manually using standard digital calipers. (D) Comparisons of manual (X-axis) and radiographic measurements (Y-axis) of the tibia showed strong correlation (R2 = 0.91). The right shift of the linear regression from the line of identity (red line) indicates that radiographic methods are systematically lower than manual readings. Since the slope of the linear regression line is not significantly different from 1.0 (1.02 ± 0.06; p = 0.66), radiographic measurements were considered a reliable indicator of differences in appendicular bone length. (E,F) Serial measurement of tibial length (N = 4) in weekly radiographs of WT C57BL/6 mice from 3 to 20 weeks of age demonstrated significant growth deceleration around the onset of puberty (7–8 weeks of age). (G) Measurements of the spinal length determined from lateral radiographs were plotted on the Y-axis against manual (caliper) measurements on the X-axis (red line, line of identity). Radiographic measurements were markedly shorter than those obtained from caliper-based methods because of the extraspinal length (skull and sacrum) included in caliper measurements. Thus, the slope of the linear regression line is significantly <1.0 (0.53 ± 0.04; p < [file Image_1.TIF]

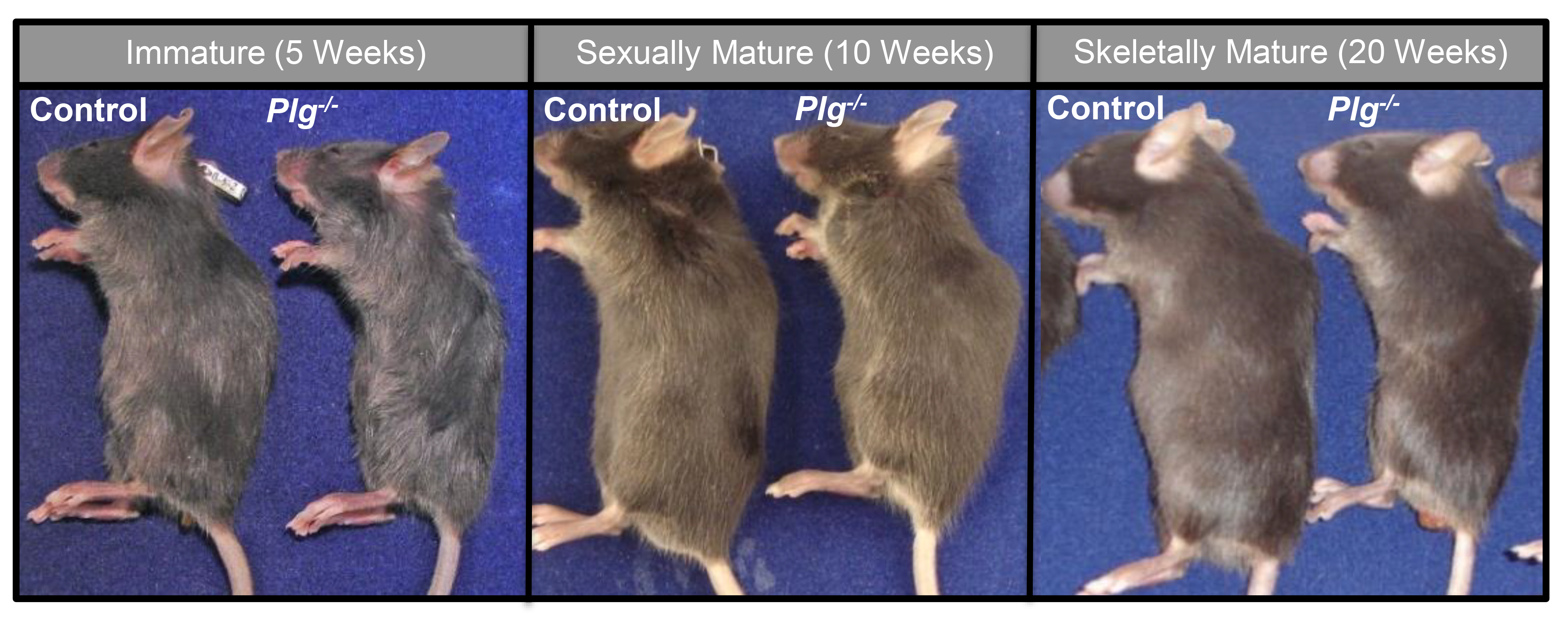

Supplement: Supplementary Figure 2 — Differences in gross postnatal development of wild type and plasminogen-deficient mice. Gross examination of Plg−/− mice and WT littermate controls demonstrated normal axial length and overall size during the immature growth phase (5 weeks of age), but stunted growth in sexually mature (10 weeks of age) and skeletally mature animals (20 weeks of age). [file Image_2.TIF]
