# Supplementary material for: The Deleterious Effects of Impaired Fibrinolysis on Skeletal Development Are Dependent on Fibrin(ogen), but Independent of Interlukin-6
Source: Front Cardiovasc Med. 2021 Dec 6;8:768338. doi: 10.3389/fcvm.2021.768338 (PMC8685342; doi:10.3389/fcvm.2021.768338)
Supplement: Supplementary Table 1 — Number of mice quantified per skeletal development measure per week. WT, Plg−/−, Fbg−/−, Plg−/−Fbg−/−, IL-6−/−, and Plg−/−IL-6−/− mice were analyzed for axial length, appendicular length, and weight each week following weaning at 3 weeks post-birth. The number of mice evaluated at each time point for each output is presented. [file Table_1.DOCX]

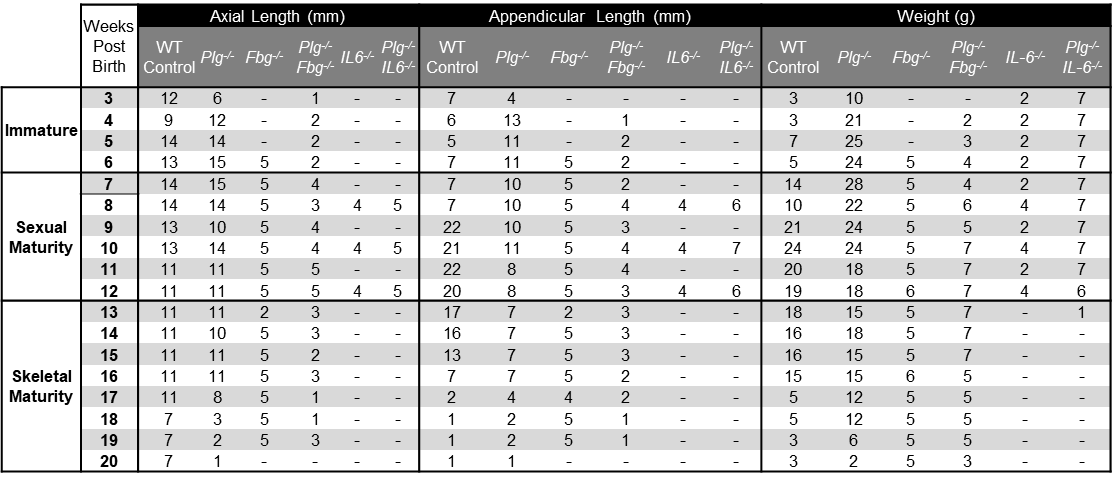


**Supplemental Table 1: Number of mice quantified per skeletal development measure per week**. WT, *Plg^-/-^, Fbg^-/-^, Plg^-/-^Fbg^-/-^, IL-6^-/-^, and Plg^-/-^IL-6^-/-^* mice were analyzed for axial length, appendicular length, and weight each week following weaning at 3 weeks post-birth. The number of mice evaluated at each time point for each output is presented.
