# Supplementary material for: The Deleterious Effects of Impaired Fibrinolysis on Skeletal Development Are Dependent on Fibrin(ogen), but Independent of Interlukin-6
Source: Front Cardiovasc Med. 2021 Dec 6;8:768338. doi: 10.3389/fcvm.2021.768338 (PMC8685342; doi:10.3389/fcvm.2021.768338)
Supplement: Supplementary Table 2 — Quantification and analysis of growth curves mice at immature (5 weeks), sexually mature (10 weeks), and skeletally mature (20 weeks) growth phases. Statistical significance between genotypes was calculated by linear regression and analysis for covariance between groups (ANCOVA). Rate of Growth: Analysis of growth curves was conducted to investigate statistical differences between the slope of the linear regression across the immature, sexually mature, and skeletally mature growth phases. In cases where differences between slopes were non-significant, absolute measurements (and therefore the Y-intercepts) were analyzed. In cases where differences between slopes were significant, it was not possible to test whether the intercepts differed significantly. Statistical analysis was conducted on the rate of axial growth, appendicular growth, and weight gain. [file Table_2.DOCX]

**
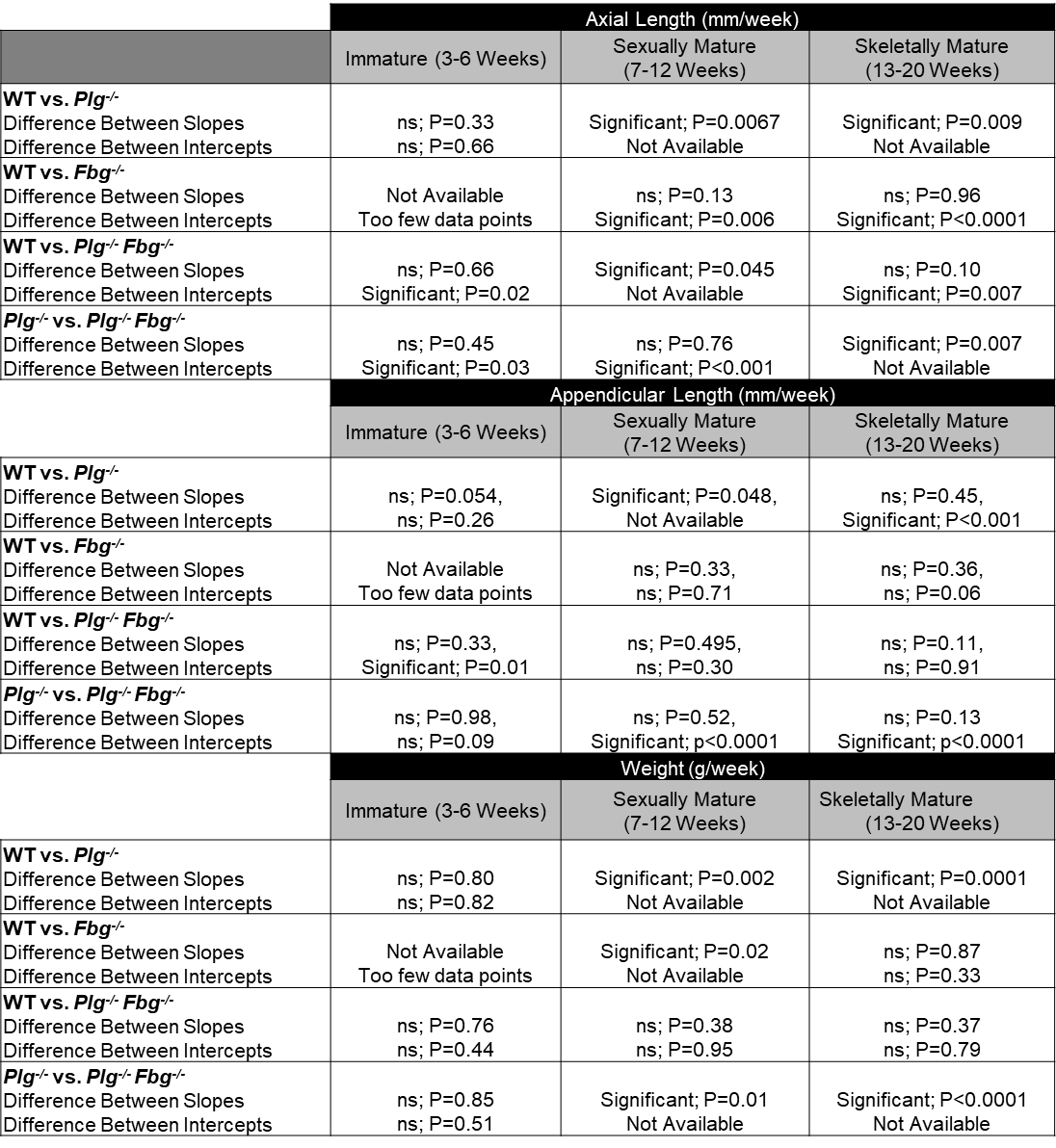
Supplemental Table 2: Quantification and analysis of growth curves mice** **at immature (5 weeks), sexually mature (10 weeks), and skeletally mature (20 weeks) growth phases**. Statistical significance between genotypes was calculated by linear regression and analysis for covariance between groups (ANCOVA). Rate of Growth: Analysis of growth curves was conducted to investigate statistical differences between the slope of the linear regression across the immature, sexually mature, and skeletally mature growth phases. In cases where differences between slopes were non-significant, absolute measurements (and therefore the Y-intercepts) were analyzed. In cases where differences between slopes were significant, it was not possible to test whether the intercepts differed significantly. Statistical analysis was conducted on the rate of axial growth, appendicular growth, and weight gain.
