# Supplementary material for: The Deleterious Effects of Impaired Fibrinolysis on Skeletal Development Are Dependent on Fibrin(ogen), but Independent of Interlukin-6
Source: Front Cardiovasc Med. 2021 Dec 6;8:768338. doi: 10.3389/fcvm.2021.768338 (PMC8685342; doi:10.3389/fcvm.2021.768338)
Supplement: Supplementary Table 3 — Histomorphometic Analysis of the Secondary Spongiosa of Distal Femurs from 10-week-old male Plg−/− and WT control mice. Values represent mean ± SD. All analyses were statistically evaluated using unpaired t-tests with multiple comparisons and a false discovery rate of 5%. [file Table_3.DOCX]

| **Supplemental Table 3: Histomorphometric Analysis** | | |
| --- | --- | --- |
| **Parameter** | **WT** | ***Plg^-/-^*** |
| Percent mineralization surface/bone surface (MS/BS) % | 31.4 ± 4.8 | 21.8 ± 2.8* |
| Bone formation rate/bone surface (BFR/BS) µm^3^/µm^2^/d | 204.7 ± 92.9 | 252.4 ± 90.2 |
| Bone formation rate/bone volume (BFR/BV) % | 1300 ± 639 | 1692 ± 1286 |
| Trabecular bone volume/total tissue volume (BV/TV) % | 6.0 ± 1.7 | 4.2 ± 0.3 |
| Trabecular thickness (Tb.Th) µm | 31.9 ± 3.4 | 29.2 ± 1.0 |
| Trabecular Number (Tb.N) #/mm | 1.9 ± 0.4 | 1.4 ± 0.2 |
| Trabecular Space (Tb. Sp) µm | 524.1 ± 114.0 | 672 ± 94.2 |
| Percent double labeled surface/ bone surface (dLS/BS) % | 9.9 ± 5.3 | 1.3 ± 1.5* |
| Percent single labeled surface/ bone surface (sLS/BS) % | 21.5 ± 1.6 | 17.4 ± 3.8 |
| Number of Osteoblasts (N.Ob) | 117.3 ± 66.7 | 64.7 ± 29.6 |
| Percentage of osteoblast surface/ bone surface (Ob.S/BS) % | 6.8 ± 3.2 | 6.6 ± 2.2 |
| Number of osteoblasts per bone perimeter (N.Ob/B.Pm) #/mm^2^ | 7.6 ± 3.0 | 7.5 ± 2.3 |
| Number of osteoclasts (N.Oc) | 31.0 ± 7.9 | 37.8 ± 16.5 |
| Percentage of osteoclast surface/ bone surface (Oc.S/BS) % | 4.9 ± 1.2 | 10.7 ± 1.9* |
| Number of osteoclasts per bone perimeter (N.Oc/B.Pm) #/mm^2^ | 2.1 ± 0.5 | 4.2 ± 0.5* |
| p<0.05, * | | |

**Supplemental Table 3: Histomorphometic Analysis of the Secondary Spongiosa of Distal Femurs from 10-week-old male *Plg^-/-^* and WT control mice.** Values represent mean ± SD. All analyses were statistically evaluated using unpaired t tests with multiple comparisons and a false discovery rate of 5%.
